# Supplementary material for: Correlations in the Binding Energy of Triexcitons and Biexcitons in Single CdSe/CdS Nanoplatelets Revealed by Heralded Spectroscopy
Source: J Phys Chem Lett. 2025 Oct 21;16(43):11267–73. doi: 10.1021/acs.jpclett.5c02589 (PMC12581158; doi:10.1021/acs.jpclett.5c02589)
Supplement: Supplementary file 1 [file jz5c02589_si_001.pdf]

Supporting Information: Correlations in the Binding Energy of Triexcitons and Biexcitons in Single CdSe/CdS Nanoplatelets Revealed by Heralded Spectroscopy

Daniel Amgar,[1] *Dekel Nakar,*[1] *Nadav Frenkel,*[1] *and Dan Oron*[1,*]

[1] Department of Molecular Chemistry and Materials Science, Weizmann Institute of Science, Rehovot 76100, Israel

S1: Characterization of the CdSe/CdS nanoplatelets

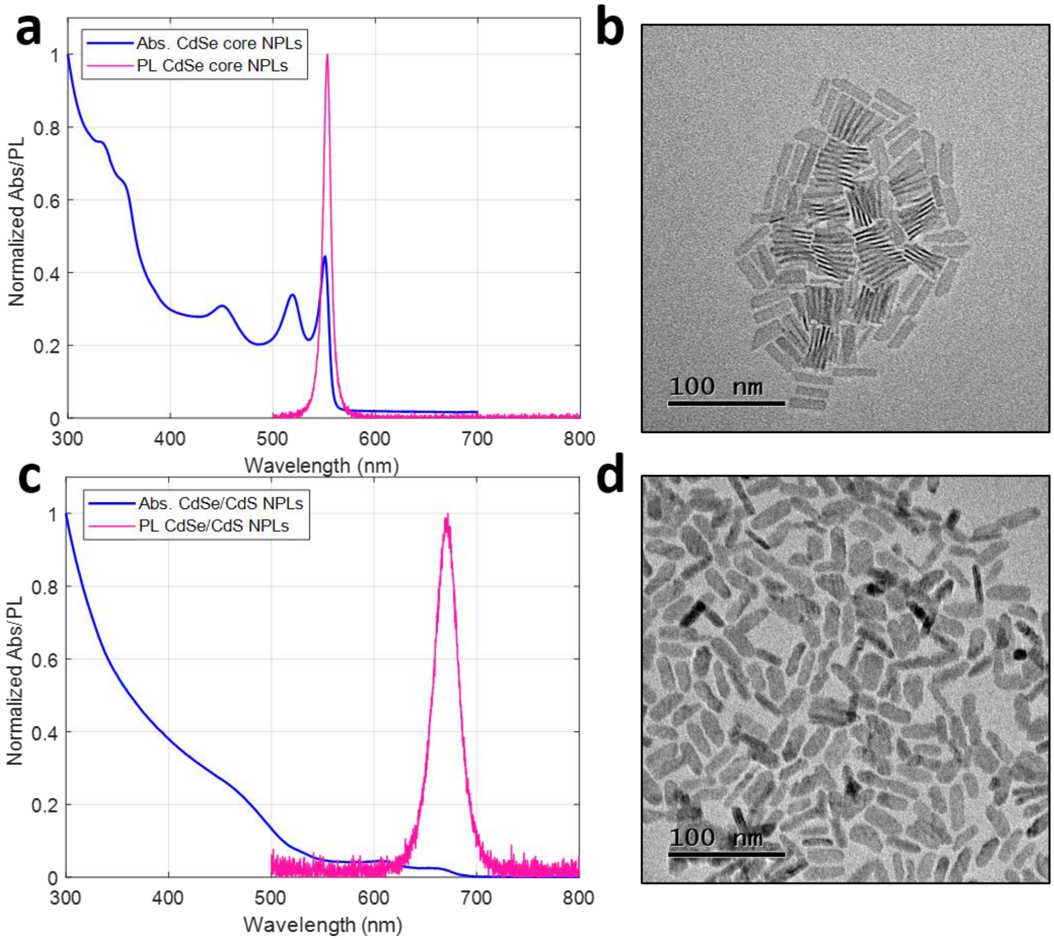

Figure S1. (a) Normalized absorption (Abs, blue) and photoluminescence (PL, pink) of the 5-monolayer CdSe core NPLs. (b) A transmission electron microscope (TEM) image of the CdSe core NPLs. (c) Normalized absorption (blue) and PL (pink) of the CdSe (5 monolayers)/CdS (3 monolayers) core/shell NPLs. (d) A TEM image of the CdSe/CdS core/shell NPLs.

Characterization methods. TEM images were taken on a JEOL 2100 TEM equipped with a LaB6 filament at an acceleration voltage of 200 kV on a Gatan US1000 CCD camera. UV-vis absorption spectra were measured using a UV-vis-NIR spectrometer (V-670, JASCO). Photoluminescence spectrum was measured using USB4000 Ocean Optics spectrometer excited by a fiber-coupled 407 nm LED in an orthogonal collection setup.

S2: Saturation assay

In order to determine the suitable laser power to be used in the experiments, a single-particle saturation measurement was performed, as described at length in the supporting information (SI) of ref [1]. Briefly, a single particle was illuminated with increasing laser power from ~35 nW to ~500 nW, in 15 equally-spaced power steps of ~30 nW, where each power step was measured for 10 seconds. Then, the power was decreased from the maximal power back to the starting power in a similar stepwise manner so that overall the particle was measured at each power for 20 seconds (10 seconds in the ascent and another 10 seconds in the descent). A saturation curve was constructed, as shown in figure S2 and described in the SI of refs [1,2]. The curve was fitted to a saturation function, extracting a nominal saturation power of 132 nW, and the actual power used in all measurements was 200 nW (nominally 205 nW, due to a 5 nW deviation between the calibrated power in the measurement of figure S2 to the actual power measured at the back entrance of the microscope with a power meter), which is beyond saturation. This power was used in order to enable measuring a sufficient number of photon triplets, assuming a negligible number of photon quadruplets. The probability to absorb at least n photons per excitation pulse can be estimated from the Poissonian distribution, giving probabilities of ~79%, ~46%, ~20%, and ~7% for absorbing at least one, two, three, and four photons, respectively. The average number of absorbed photons per pulse ($\langle N \rangle$) can be calculated by the power used (I_{used}) divided by the extracted saturation power (I_{sat}): ${}^{I_{used}}/_{I_{sat}}$, which in this case is $\sim 1.6 \pm 0.2$. We note that due to the non-negligible $g^{(2)}(0)$, this saturation is that of emitted photons, rather than absorbed photons, meaning that at I_{sat}, more than one photon is absorbed per pulse.

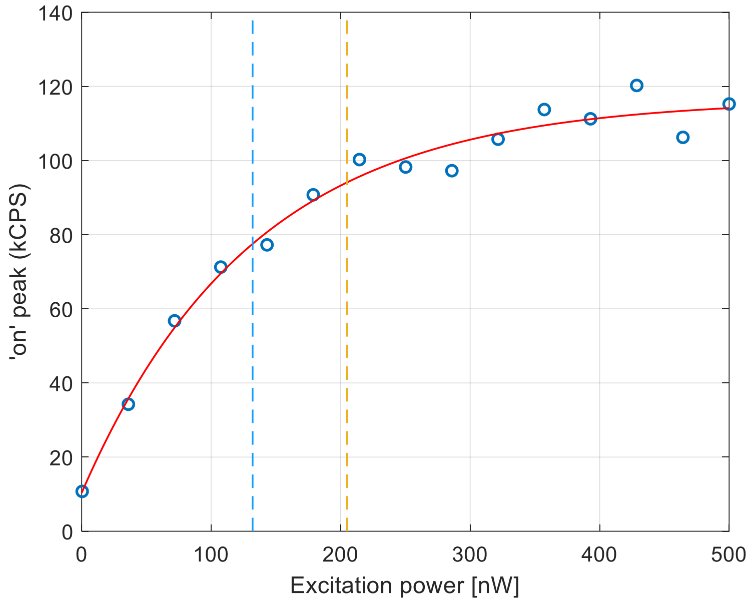

Figure S2. Saturation curve measured for a single nanoplatelet. The orange dashed line in 205 nW indicates the nominal power used in this measurement and the blue dashed line at 132 nW indicates the saturation power extracted from the fit to a saturation function (red curve).

S3: Apparatus

The spectroSPAD system is practically a home-built single-photon spectrometer. A 470 nm pulsed laser (70 ps, LDH-P-C-470B, PicoQuant) with repetition rate of 5 MHz enters the back of an inverted optical microscope (Eclipse Ti-U, Nikon), reflected by a 484 long-pass dichroic mirror (FF484-FDi02-t3, Semrock), and focused on a single particle in the sample through a high NA x100 oil-immersion objective lens (1.3 NA, Nikon). The epi-detected photoluminescence (PL) emitted from the single particle is then collected via the same objective, passes the dichroic mirror and a 473 nm long-pass emission filter (BLP01-473R, Semrock) in order to filter out the 470 nm excitation laser. The image plane of the microscope is the input of a Czerny-Turner spectrometer based of a 4f system (AC254-300-A-ML and AC254-100-A-ML, Thorlabs) and a blazed diffraction grating (53-*-426R, Richardson Gratings). The output of the spectrometer is coupled to a monolithic linear single-photon avalanche diode (SPAD) array detector (synchronized with the laser), which is composed of 512 pixels with a pixel pitch of 26.2 μm (spectral resolution is ~1.7 nm), out of which 64 pixels are connected to a time-to-digital converters (TDCs) implemented by a field-programmable gate array (FPGA). This module registers the time stamp and pixel of each detected photon so that finally a list of photons along with their pixel addresses and arrival times (given by the pulse number after which the photon has arrived and the time delay from the beginning of that pulse). Due to excessive dark counts in pixel 34, we excluded it from the analysis. Further details are found in refs [1,3].

S4: Lifetime analysis of a single nanoplatelet (NPL)

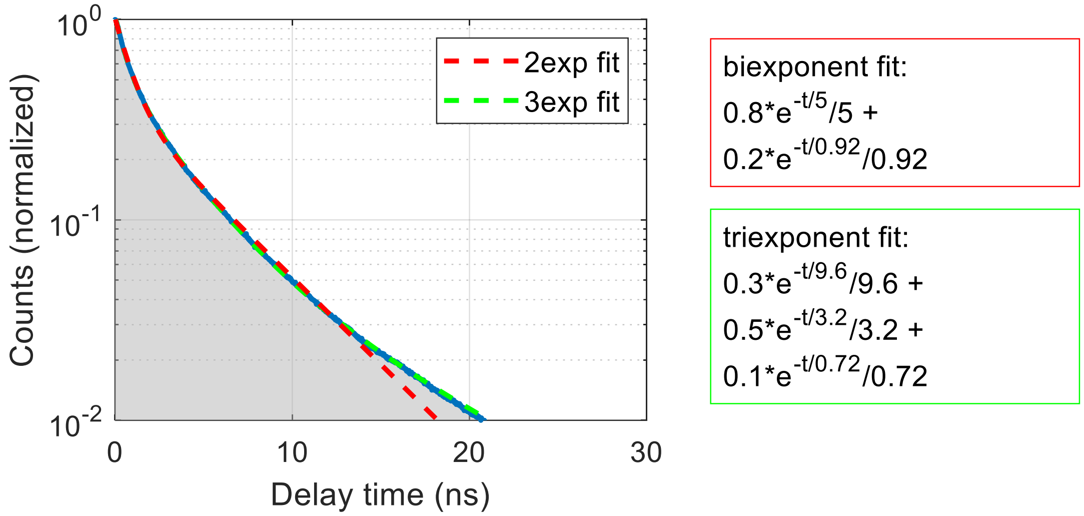

Figure S3. Lifetime curve (blue solid line) of a single NPL extracted by full vertical binning of figure 2b of the main text, along with a biexponential (dashed red line) and triexponential (dashed green line) fits. The respective fitting results are presented on the right side, yielding lifetimes of 5 ns and 0.92 ns from the biexponential fit and lifetimes of 9.6 ns, 3.2 ns, and 0.72 ns from the triexponential fit.

S5: A magnified image of the third- versus second-order photon correlation

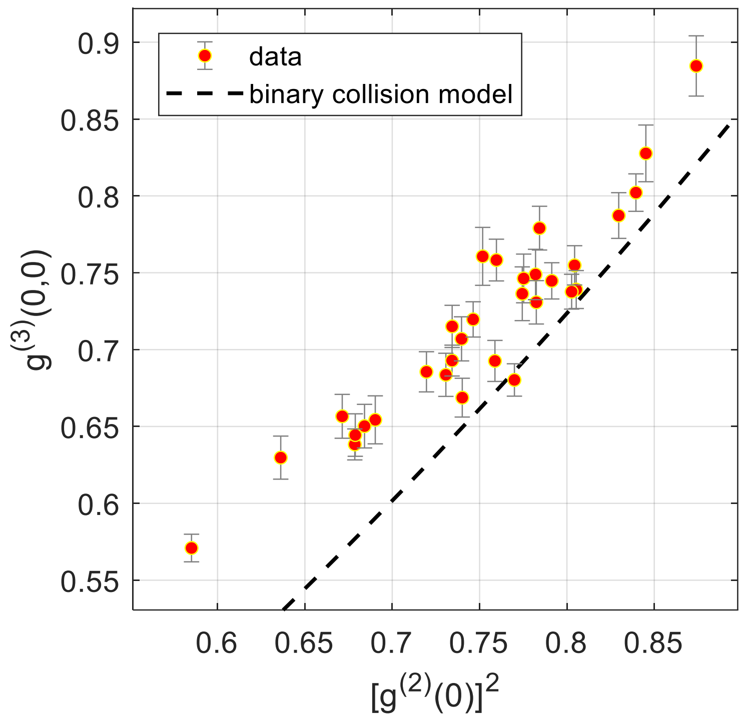

Figure S4. A magnified view of the datapoints in figure 3 of the main text. Third-order correlation at time delays of zero between the three detections $[g^{(3)}(0,0)]$ versus second-order correlation at zero time delay squared $\{[g^{(2)}(0)]^2\}$ for all measured NPLs. The black dashed line represents $g^{(3)}(0,0)$ calculated through the binary collision model (see details in ref [4]).

S6: Heralded spectra spanning all of the detector spectral range

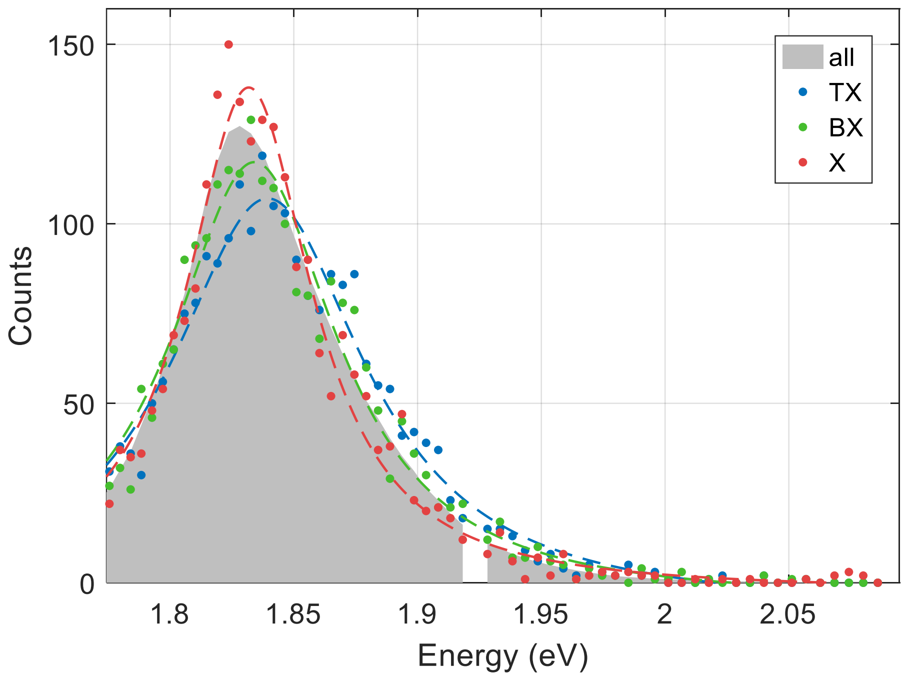

Figure S5. Single NPL spectra of the triexciton, TX (blue), biexciton, BX (green), and exciton, X (red) photons. The blue, green, and red dashed lines are fits of the TX, BX, and X spectra, respectively, to a Cauchy-Lorentz distribution. The gray area corresponds to all detected photons, without heralded post-selection, normalized to match the TX counts. The nullified data at ~1.93 eV is due to the removal of the noisy pixel. This plot corresponds to figure 4a of the main text, displaying a zoomed-out view that reveals the full spectral range detectable by the spectroSPAD system.

S7: Additional examples of single-nanoplatelet analysis

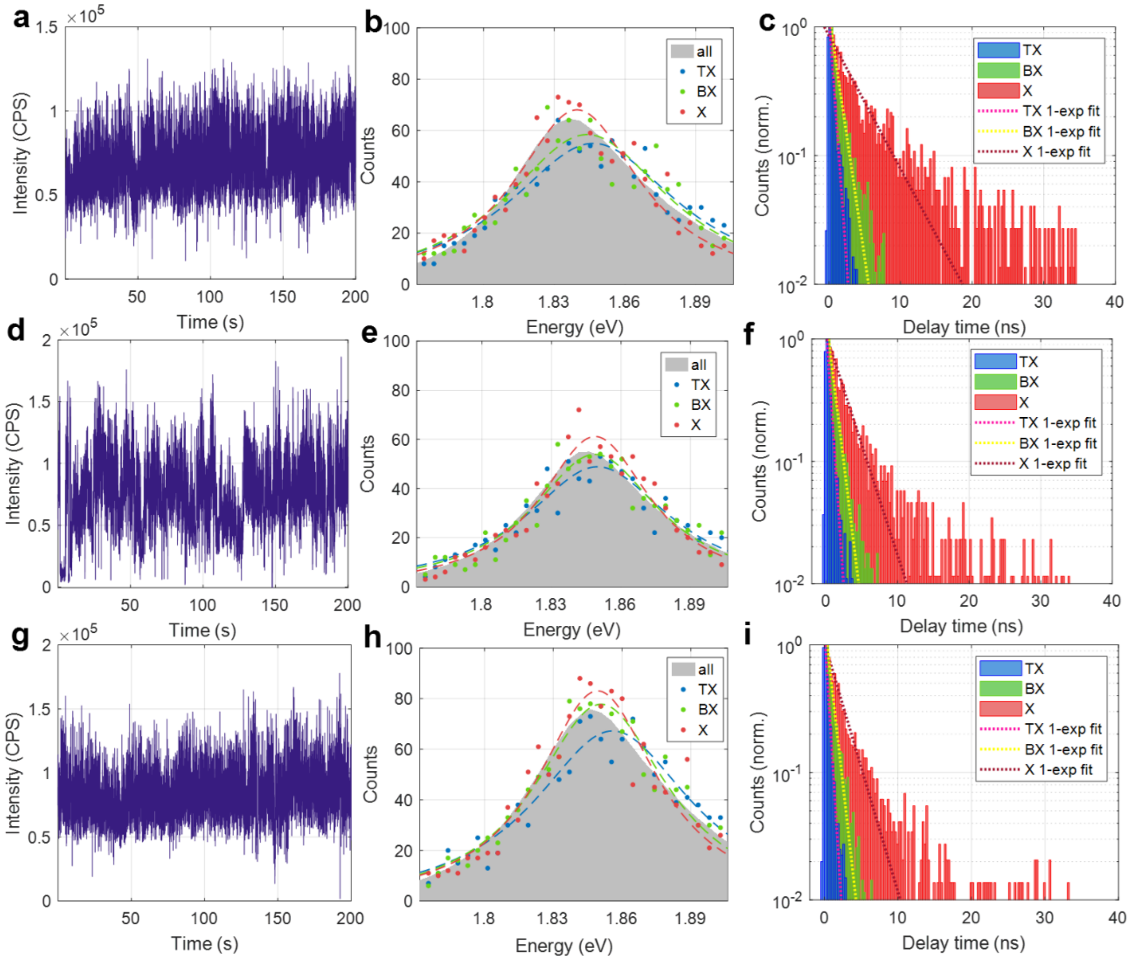

Figure S6. Other examples of single-NPL analysis. Panels a–c correspond to NPL1; Panels d–f correspond to NPL2; Panels g–i correspond to NPL3. (a,d,g) Intensity trace for a 200-seconds acquisition time window for the three different NPLs. (b,e,h) Single NPL spectra of the triexciton (blue), biexciton (green), and single exciton (red) photons for the three NPLs. The blue, green, and red dashed lines are fits of the triexciton, biexciton, and single exciton spectra, respectively, to a Cauchy-Lorentz distribution. The gray area corresponds to all detected photons, without heralded post-selection, normalized to match the triexciton counts. (c,f,i) Lifetime histograms of the three single NPLs along with monoexponential fits. The delay time of the triexciton is from the laser peak, and the delay times of the biexciton and single exciton are from the preceding arrival times of the triexciton and biexciton, respectively.

S8: Second-order correlation versus time gating of photon detections[4]

The gated $g^{(2)}$ assay was done by gating the time delay of photons relative to the previous laser pulse and using only late-arriving photons to construct the $g^{(2)}(\tau)$ curve and calculate the gated $g^{(2)}(0)$. The chosen time gate applied for all NPLs is from 6 to 35 ns, meaning that only photons with time delays higher than 6 ns and lower than 35 ns are considered for the $g^{(2)}$ analysis. The lower limit of 6 ns aims to remove the contribution from multiexcitons, which relax faster, and the upper limit of 35 ns aims to remove noise. In this way, late-arriving photons should represent photons emitted from the single exciton state only, expected to increasing the degree of antibunching (reducing $g^{(2)}(0)$).[4,5] Figure S7 shows the degree of antibunching, the reduced probability of detecting two photons at the same laser period, versus gating times. Gating the photons at 3 ns already shows a decrease of the $g^{(2)}(0)$ below 0.5, which is taken as a criterion for a single emitter, meaning measuring only a single NPL at a time.[6]

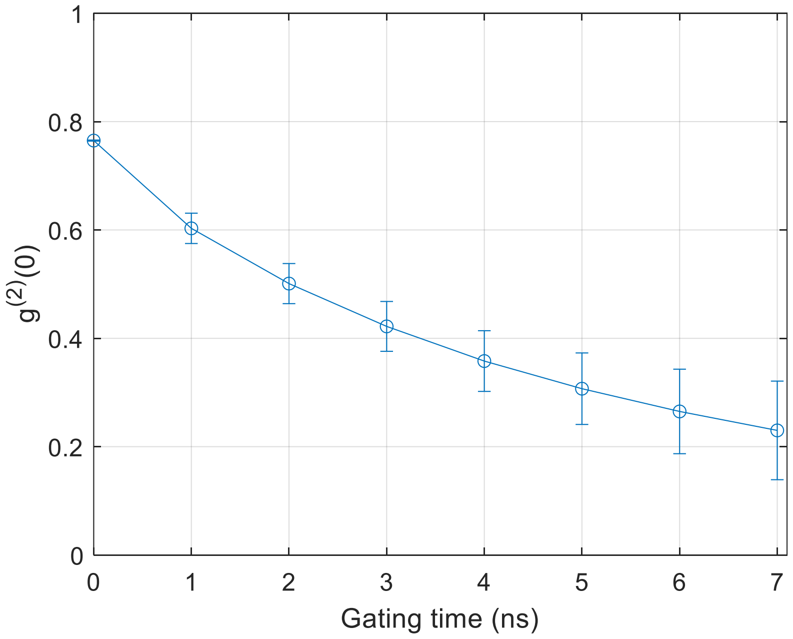

Figure S7. Second-order photon correlation values at zero time delay ($g^{(2)}(0)$) for increasing gating times in a single NPL. The $g^{(2)}(0)$ shows a decrease (i.e., increasing antibunching) with increasing the gating time, down to about 0.2 for a gating time of 7 ns.

S9: CdSe/CdS NPLs with varied thicknesses

As a complementary experiment, we synthesized four CdSe/CdS NPLs samples with a growing number of CdS shell monolayers (ML) using the method of colloidal atomic layer deposition (c-ALD), as described in ref 7. This process was repeated several times up to the desired number of shell MLs. We note that the CdSe NPL cores used for these syntheses are somewhat smaller than the ones used for all other results reported in the manuscript and SI. We synthesized NPLs with one, two, three, and four CdS MLs. Normalized photoluminescence spectra of the samples are plotted in figure S8a, showing a gradual redshift of the spectrum with increasing the shell thickness due to the strong quantum confinement in the thickness dimension. Single-NPL measurements were performed with the spectroSPAD system for each sample to estimate the biexciton (BX) binding energies. Figure S8b summarizes the BX binding energies as a function of the exciton (X) peak, estimated through heralded spectroscopy, and clearly shows a transition from attractive to repulsive BX binding energies as the thickness is increased. Additionally, few single CdSe NPL cores were measured (both the ones used in this assay and the others used in all other reported results) and are not presented here due to low BX count. Single core NPLs are highly difficult to measure due to low stability under illumination. The mean value of the BX binding energies estimated for all CdSe NPL cores was -7.8 ± 1.4 meV, pointing at attractive BX binding energy, as anticipated. It should be noted that these single NPLs (both core and core/shell) could not yield enough TX detections to further estimate the TX binding energies, yet it is plausible to assume that the TX binding energies would have been repulsive as well.

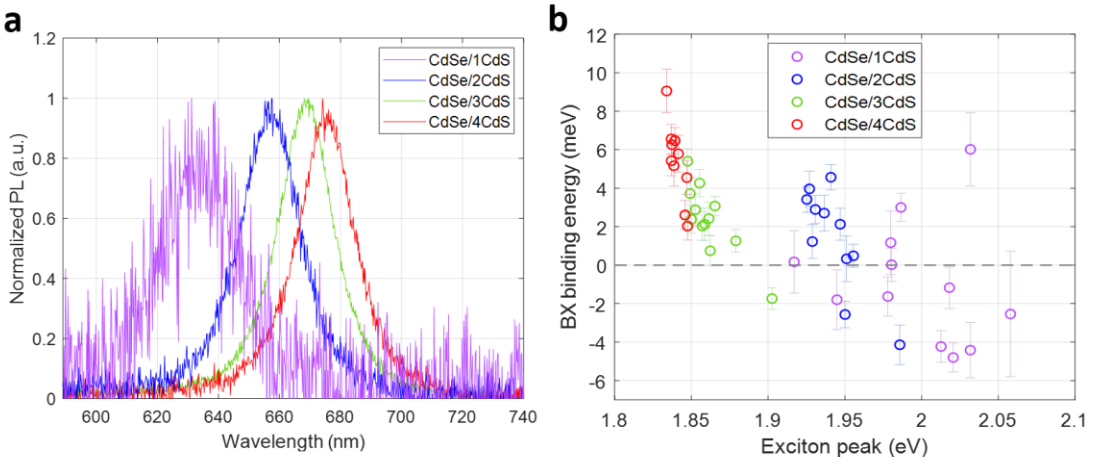

Figure S8. (a) Normalized photoluminescence of four NPL samples with different number of CdS shell monolayers. (b) Biexciton binding energies versus the exciton peak estimated through the heralded analysis for single-NPL measurements of four NPL samples with different number of CdS monolayers as a shell. Purple, blue, green, and red colors correspond to one, two, three, and four CdS shell monolayers.

S10: Correlation of the triexciton and biexciton binding energies

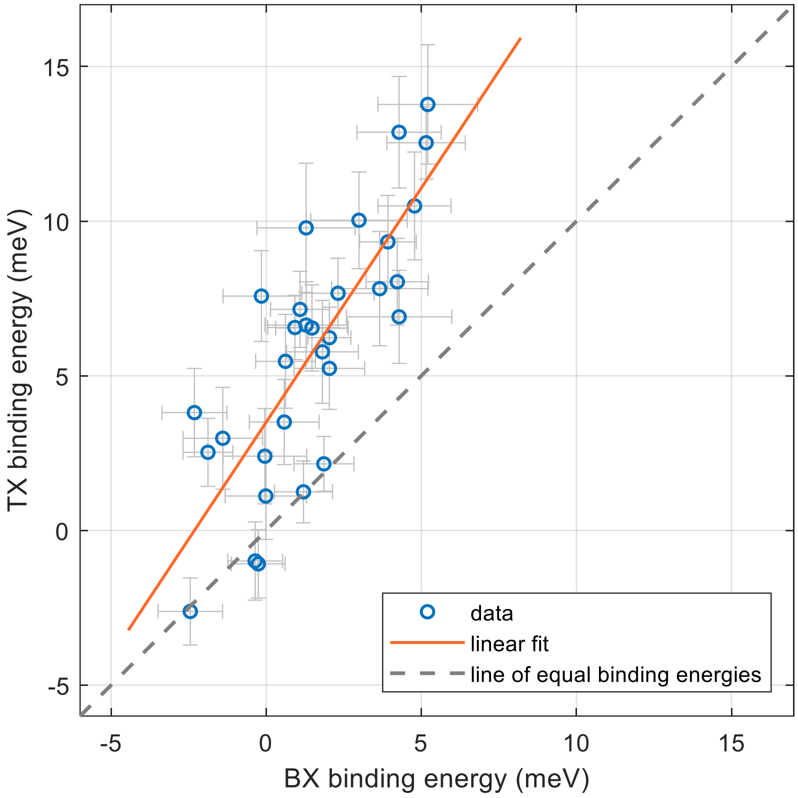

Figure S9. Correlation of the triexciton (TX) and biexciton (BX) binding energies of all measured single NPLs. The orange solid line is a linear fit to the data. The gray dashed line is a guide to the eye, indicating identical TX and BX binding energies.

References

1. Lubin, G. *et al.* Heralded Spectroscopy Reveals Exciton–Exciton Correlations in Single Colloidal Quantum Dots. *Nano Lett* **21**, 6756–6763 (2021).

2. Frenkel, N. *et al.* Two Biexciton Types Coexisting in Coupled Quantum Dot Molecules. *ACS Nano* **17**, 14990–15000 (2023).

3. Lubin, G. *et al.* Resolving the Controversy in Biexciton Binding Energy of Cesium Lead Halide Perovskite Nanocrystals through Heralded Single-Particle Spectroscopy. *ACS Nano* **15**, 16 (2021).

4. Amgar, D., Yang, G., Tenne, R. & Oron, D. Higher-Order Photon Correlation as a Tool To Study Exciton Dynamics in Quasi-2D Nanoplatelets. *Nano Lett* **19**, 8741–8748 (2019).

5. Benjamin, E. *et al.* Temperature Dependence of Excitonic and Biexcitonic Decay Rates in Colloidal Nanoplatelets by Time-Gated Photon Correlation. *Journal of Physical Chemistry Letters* **11**, 6513–6518 (2020).

6. Grußmayer, K. S. & Herten, D.-P. Photon Antibunching in Single Molecule Fluorescence Spectroscopy. In: Kapusta, P., Wahl, M., Erdmann, R. (eds) Advanced Photon Counting. Springer Series on Fluorescence, **15**, Springer, Cham., 159–190 (2014).

7. Yang, Z., Pelton, M., Fedin, I., Talapin, D. V & Waks, E. A room temperature continuous-wave nanolaser using colloidal quantum wells. *Nat Commun* **8**, 1-8 (2017).
